# Differential gene expression induced by anti-cancer agent plumbagin is mediated by androgen receptor in prostate cancer cells

**DOI:** 10.1038/s41598-018-20451-9

**Published:** 2018-02-09

**Authors:** Gaelle Rondeau, Parisa Abedinpour, Adrian Chrastina, Jennifer Pelayo, Per Borgstrom, John Welsh

**Affiliations:** 1 Vaccine Research Institute of San Diego, 3030 Bunker Hill Street, Suite 200, San Diego, CA 92109 USA; 2 Pellficure Pharmaceuticals, Inc., 2325 Camino del Collado, La Jolla, CA 92037 USA

## Abstract

Treatment of mice harboring PTEN-P2 tumors in the prostate or on prostate tissue *in vivo* with 5-hydroxy-2-methyl-1,4-naphthoquinone, also known as plumbagin, results in tumor regression in castrated mice, but not in intact mice. This suggested that dihydrotestosterone (DHT) production in the testes may prevent cell death due to plumbagin treatment, but the underlying mechanism is not understood. We performed RNA-seq analysis on cells treated with combinations of plumbagin and DHT, and analyzed differential gene expression, to gain insight into the interactions between androgen and plumbgin. DHT and plumbagin synergize to alter the expression of many genes that are not differentially regulated by either single agent when used alone. These experiments revealed that, for many genes, increases in mRNAs caused by DHT are sharply down-regulated by plumbagin, and that many transcripts change in response to plumbagin in a DHT-dependent manner. This suggests that androgen receptor mediates some of the effects of plumbagin on gene expression.

## Introduction

Plumbagin (5-hydroxy-2-methyl-1,4-naphthoquinone) is a small molecule derived from the Plumbaginaceae family, including Plumbago indica and Plumbago zelanica^1,2^, certain carnivorous plants of the Droseraceae family^3^, plants of the Ebenaceae family^4^, wherein it is thought to protect against predation^3^, and in species of the Juglans genus^5^. It has been used in traditional medicine for various purposes, and has been studied more recently as a potential cancer therapeutic^5–13^. Plumbagin prevents growth of androgen-independent prostate cancer cells in xenografts, and invasion in cell culture experiments^8^.

Plumbagin has three properties that may influence gene expression. Plumbagin can engage in Michael addition, which results in arylation of nucleophiles, including thiols and amines, in circulation and in the cell. This activity explains the rapid clearing of free plumbagin from the circulation^14^. Other quinones are known to induce endoplasmic reticulum stress via their Michael addition property, with attendant changes in gene expression^15^. Plumbagin also engages in redox recycling that generates reactive oxygen species (ROS). Plumbagin can exchange electrons with NADH and NADPH using enzyme catalysis, possibly using POR (cytochrome p450 oxidoreductase) or NQO1 (NAD(P)H-quinone dehydrogenase 1)^16^. In its semiquinone state, plumbagin can reduce oxygen to produce superoxide, which can be converted to hydrogen peroxide in a reaction catalyzed by superoxide dismutase. Both superoxide and peroxides can damage proteins, nucleic acids, and other molecular components in the cell^17^, suggesting a complex initial cellular response to plumbagin. A third mechanism, wherein plumbagin binds directly to active sites of enzymes, has also been explored^18^.

Androgen dependent PTEN-P2 cells were derived from a heterozygous null mutant for the Pten gene^19,20^. Pten^−/+^ mice develop prostatic interstitial neoplasia (PIN) after long latency, which do not progress to carcinoma^20^. However, loss of both Pten alleles in conditional knockout mice leads to PIN lesions that progress to invasive carcinoma^20^. Also, when Pten^−/+^ was coupled with Cdkn1b^−/−^ (aka p27Kip1^−/−^), prostate carcinoma developed within 3 months of birth, with complete penetrance^21^. Whereas PTEN-P2 cells do not grow in subcutaneous xenografts, PTEN-P2 cells form tumors when implanted orthotopically in nude mice^22^. These PTEN-P2 tumors are androgen sensitive, in that growth slows dramatically upon castration^6^. Therefore, we used PTEN-P2 cells implanted in the prostate and cultured *in vivo* in dorsal skin fold chambers as a model of pre-invasive prostate cancer.

In this study, we develop an overview of the genetic subsystems affected by plumbagin and DHT in PTEN-P2 cells using RNA-seq and bioinformatics. Consistent with other broadly focused studies^18^, and with largely indiscriminate effects due to free radical damage, RNA-seq data show that diverse subsystems of genes respond to plumbagin. We found that many genes that were up-regulated by DHT were down-regulated by plumbagin. Interestingly, many other genes that were not up-regulated by DHT were down-regulated by plumbagin, but only in combination with DHT, suggesting activation of a repressive mode of Ar. The suppression of normal androgen functions by plumbagin may be a component of its anti-prostate cancer properties *in vivo*. RNA-seq data also suggested that RNA metabolism is dramatically altered by plumbagin treatment. Therefore, we examined RNA integrity directly, and found that plumbagin treatment causes extensive RNA damage that increases with increasing plumbagin concentration.

## Materials and Methods

### Biologicals

Dihydrotestosterone (DHT) and plumbagin from *Plumbago indica* were purchased from Sigma-Aldrich (Saint-Louis, MO, USA). Both were dissolved in DMSO (Dimethyl Sulfoxide). PTEN-P2 mouse prostate cancer cells were provided by the Wu laboratory^19,20^, and were modified to express H2B-GFP as described previously^6^.

### Cell Culture

PTEN-P2 cells were maintained in DMEM with phenol red (Dulbecco’s Modified Eagle’s Medium) containing 4.5 g/L glucose, 10% FBS (Omega Scientific, Tarzana, CA, USA), 100 U/ml penicillin, 100 μg/ml streptomycin, insulin-selenium-transferrin (5 μg/ml insulin). G418 (100 μg/ml) was included to maintain stable expression of H2B-GFP. For experiments, PTEN-P2 cells were grown in DMEM free of phenol red (Dulbecco’s Modified Eagle’s Medium) containing 4.5 g/L glucose, 10% FBS which was treated with charcoal dextran to remove hormones (Omega Scientific, Tarzana, CA, USA), 2 mM L-glutamine, 100 U/ml penicillin, 100 μg/ml streptomycin, insulin-selenium-transferrin (5 μg/ml insulin), without G418.

### Cell growth in response to plumbagin and DHT

**Figure 1:**
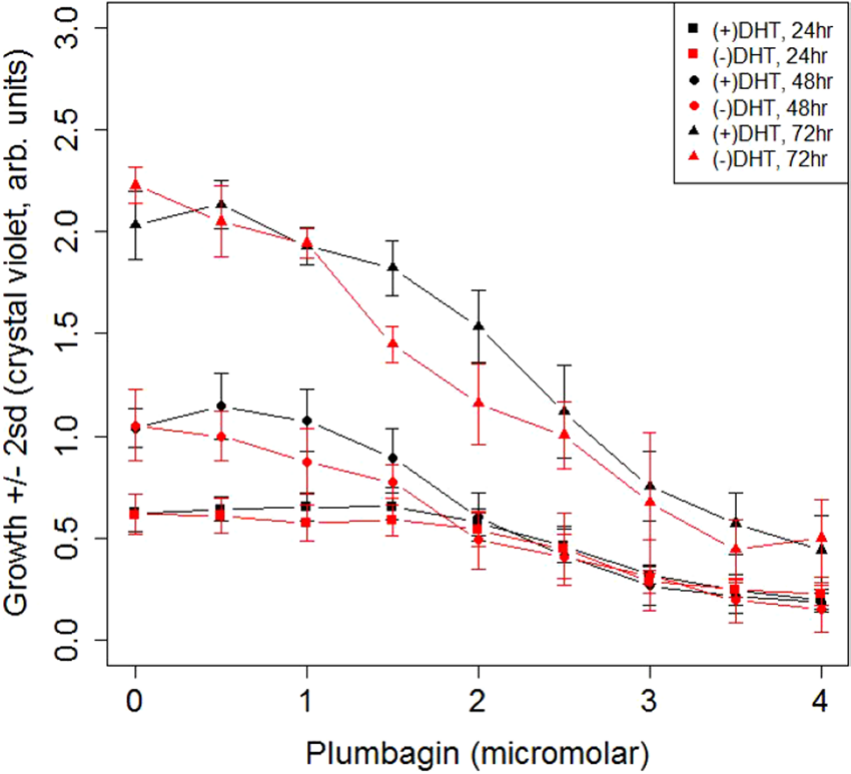
DHT treatment was for ~24 hours. Plumbagin was added to the cell culture media using DMSO as a solvent at time zero at the indicated concentrations. Cell growth was assessed using crystal violet at the indicated times. Error bars indicate two standard deviations with n = 8.

In studies of the effects of plumbagin and DHT on cell growth (Fig. 1), treatments with plumbagin are for 24, 48, 72 hr. Plumbagin was added in DMSO, equal amounts of DMSO were added to all cell cultures, and an equal amount of DMSO was added to the zero-treatment control. When DHT is present, it was added 24 hr prior to plumbagin addition, and continues throughout the growth period, i.e. up to 72 hr. without change of media. These treatments comprise a single addition of plumbagin, followed by measurement of crystal violet at the indicated times.

### Treatment with DHT and plumbagin, library preparation and sequencing

For gene expression analysis in response to plumbagin and DHT, cells in culture were treated for 1 hour with 0, 0.5, or 4 μM plumbagin dissolved in DMSO, with or without overnight and continued treatment with 10^−8^ M DHT in DMSO, or mock treatment with DMSO. Treatments were in duplicate. No-treatment controls were in triplicate. The DMSO concentration was constant between treatment conditions. After treatment, cells were harvested and RNA was purified using RNeasy (Qiagen, Germantown, MD, USA). NGS sequencing libraries were prepared from using NEBNext for Illumina® using index primers (New England Biolabs, Ipswich, MA, USA). These libraries were sequenced, 6 index primers per lane, using the Illumina HiSeq platform at the UCSD sequencing facility (http://igm.ucsd.edu/genomics). Reads were from 252–269 reads per lane.

### RNA-Seq Data Analysis

We used bowtie2^23^ and cufflinks^24^ to count sequence reads, associate them with mRNAs, and report fragments per kilobase of transcript per million mapped reads (FPKM). We removed all genes for which the average count across all samples was <1 FPKM, and all genes that produce transcripts lacking a poly-A tail, because we used oligo-dT priming to produce first strand cDNA. We used quantile normalization to map the distribution from each experimental variable to a common distribution. These data present as a two dimensional table in which each row represents an mRNA from a gene and each column represents one of the 6 combinations of +/− plumbagin (at 0.5 μM and 4 μM) and +/− DHT. Treatments were in duplicate, and no-treatment controls were in triplicate, as mentioned above. We then generated ratios between the average of the three no treatment controls and the averages of the duplicates of each treatment. We calculated false discovery rates (fdr) by determining the number of control sample differential expression ratios (i.e. ratios of expression values between replicate controls) that exceeded an experimental sample/control sample ratio, and dividing by the rank order of the experimental sample/control sample ratio. This is the fdr, i.e. fraction of genes that have higher apparent differential expression ratios by chance. 5% of genes with an fdr ≤ 0.05 are expected to be false discoveries. Only genes for which at least one condition had a differential expression ratio with a fdr ≤ 0.05 were included in further analysis. These were converted to log_2_ values. We then computed 36 k-means groups using the statistical computation programming environment, R^25^, with 10,000 starts and 1000 iterations, for mRNA frequencies averaged over all isoforms. We chose to use 36 groups subjectively, based on most of the k-means groups reflecting obvious trends (e.g. up-regulated by plumbagin in the absence but not in the presence of DHT, etc.). These data after processing can be viewed by going to www.voxvill.org/relnet.plumbagin and selecting DHT_Plumbagin under “Select a pre-loaded experiment”. Pre-processed data has been deposited with NCBI GEO (GSE104305).

### Pathway Enrichment Analysis

For identification of protein-protein interactions, we developed a web-based software platform, Gene Information Relationship Network (GIRN) (www.voxvill.org/relnet.plumbagin), that uses standard methods to identify proteins whose connections to proteins in a list are enriched, and is described in detail in (http://biorxiv.org/content/early/2016/10/07/079590). In GIRN, genes that are locally enriched are identified using a z-score calculated using a binomial proportions test, as in^26^. We used GIRN DB version 0, in which the protein-protein interaction network consists of 1,145,437 non-redundant interactions from Kamburov *et al*.^27–30^ in Consensus Path Database (CPDB) (http://consensuspathdb.org), from Cerami *et al*.^31^ in Pathway Commons (http://www.pathwaycommons.org)(Pathway Commons.7.All.EXTENDED_BINARY_SIF.hgnc.sif; downloaded 3/21/16; 914,165 interactions), and from HTRIdb (Human Transcriptional Regulation Interactions database; downloaded 3/21/16; 52,467 interactions) for the Transcription Factor-Transcribed Gene component from Bovolenta *et al*.^32^ (http://www.lbbc.ibb.unesp.br/htri). CPDB includes BioCarta (http://cgap.nci.nih.gov/Pathways/BioCarta_Pathways), EHMN, HumanCyc (http://humancyc.org), INOH, KEGG (http://www.genome.jp/kegg), NetPath (http://www.netpath.org), PharmGKB (http://www.pharmgkb.org), PID (http://pid.nci.nih.gov), Reactome (http://reactome.org), Signalink (http://signalink.org), SMPDB (http://www.smpdb.ca), and Wikipathways (http://www.wikipathways.org).

### Sub-network Annotation Enrichment Analysis

The gene ontology database used by GIRN consists of the Gene Ontology Annotation (UniProt-GOA) Database and the Gene Ontology Consortium (www.geneontology.org)^33,34^. Specifically, we used the gene_association.goa_ref_human subset (9.7.2016 release) of the UniProt-GOA database. Drug-gene interactions are from DGIdb^35^ (http://dgidb.genome.wustl.edu). Genes associated with diseases are from DisGeNET (http://www.disgenet.org)^36,37^. Pathways are from Reactome^38,39^ (http://reactome.org). Enrichment analysis used Fisher’s exact test (two-sided), and p-values were modified post-hoc for multiple testing using Bonferroni correction, yielding an estimate of the family-wise error rate (FWER). Fisher’s exact test uses a 2 × 2 contingency table that contains the number of genes in a group associated with an annotation term, the number of genes in the group not associated with the term, the total number of genes associated with the term, and the total number of genes. For Bonferroni correction, we used α/N, where N is the total number of genes.

## Results

Pten^+/−^ mice develop spontaneous tumors in multiple tissues and develop prostatic intraepithelial neoplasia (PIN) after a long latency period^19,20^. These PIN lesions do not progress to metastatic cancer, whereas mice with both Pten^+/−^ and Cdkn1b^−/−^ develop prostate carcinoma with high penetrance^21^. In this context, the PTEN-P2 cell line, which is hemizygous null for Pten, represents a model for an early event in the development of prostate cancer. In intravital microscopy (IVM) experiments, PTEN-P2 tumors stop growing upon androgen deprivation therapy (ADT) but do not regress, grow at a slower rate when treated with plumbagin alone, and regress when treated with plumbagin and ADT in combination^6^. In animals with prostate tumors, plumbagin treatment alone and ADT alone ultimately failed, but the combination therapy resulted in complete tumor regression^22^. IVM studies reveal many pyknotic structures in experimental tumors when the animals are castrated and treated with plumbagin, consistent with previous observations that cell cycle arrest due to plumbagin occurs primarily in G2/M^6^.

Figure 1 shows the effect on PTEN-P2 cells exposed to different concentrations of plumbagin over time, with and without DHT treatment. DHT has a reproducible but minor effect on cell proliferation at intermediate levels of plumbagin, and a negligible effect at lower concentrations (e.g. 0.5 μM). Single addition of 2 μM plumbagin or more inhibits cell growth for ~2 days, after which growth resumes at the normal rate. This is most evident in the ~2-fold increase in cell mass for plumbagin concentrations above 2 μM after 72 hr. While DHT regulates the sensitivity of PTEN-P2 experimental tumors to plumbagin *in vivo*, it has only minor effects on growth of PTEN-P2 cells in cell culture.

**Figure 2:**
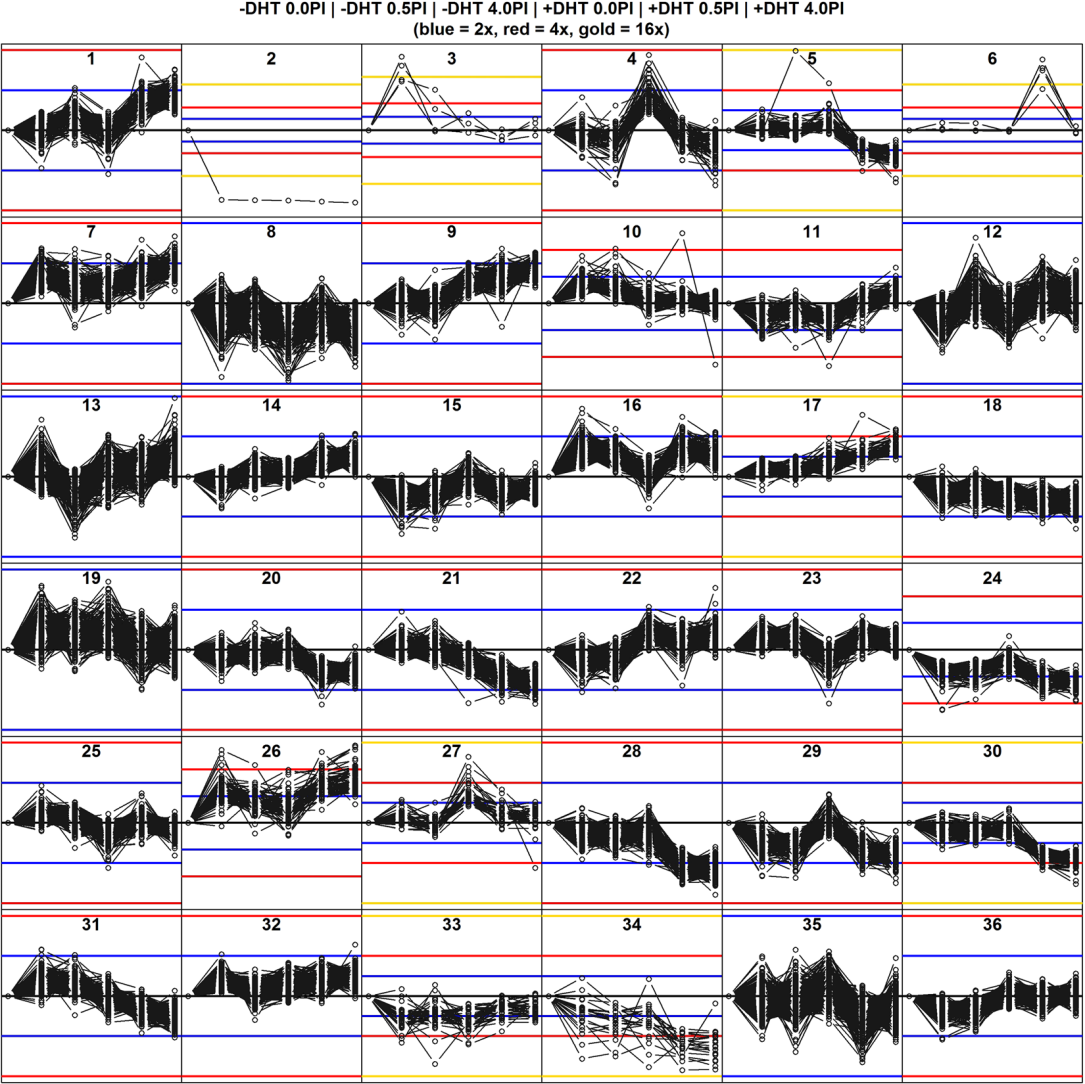
RNA-seq quantitation of mRNAs was achieved as described in Materials and Methods. K-means was applied to RNA molecule counts for 6 treatment groups plotted left to right in each of 36 boxes, respectively, (1) no treatment, (2) 0.5 μM plumbagin, (3) 4 μM plumbagin, (4) 10^−8^ M DHT, (5) 10^−8^ M DHT + 0.5 μM plumbagin, and (6) 10^−8^ M DHT + 4 μM plumbagin. Treatment with DHT was overnight, and treatment with plumbagin was for 1 hr. Gene expression responses were determined by RNA-seq, and are expressed as log_2_ ratios relative to the untreated control. Groups KM1 through KM36 are shown. Blue horizontal lines represent 2-fold changes, red lines represent 4-fold changes, and gold lines represent 16-fold changes. Note that the vertical scale, which corresponds to the log_2_ of magnitude of change relative to the untreated control (black line), varies from box to box.

We treated PTEN-P2 cells with plumbagin (0.5 μM or 4 μM), 10^−8^ M DHT, or both, and performed RNA-seq expression profiling. DHT treatment was for ~24 hr, and plumbagin treatment was for 1 hr. This short time frame and the lower concentration of plumbagin (0.5 μM) were chosen to allow us to examine immediate responses to plumbagin, and to minimize confounding effects associated with profound damage and cell death. Under these conditions, plumbagin causes hundreds of changes in gene expression within one hour of treatment, and this response often depends on prior treatment with 10^−8^ M DHT. Interaction between plumbagin and DHT is clear when RNA-seq data is resolved into different response classes using k-means. Figure 2 shows k-means grouping of 8112 genes that responded to either plumbagin, DHT, or both into 36 response classes. Several of the groups contain genes that have similar expression patterns, such as KM5, 28, and 30, in which genes show no change or small changes in expression in response to plumbagin or DHT treatment alone, but large changes in expression when the two agents were combined. Groups that contain only small variations centered around zero (KM12, 13, 19, and 35), and groups contain small numbers of genes that do not lend themselves to statistical analysis (KM2, 3, and 6), are ignored henceforth.

The K-means algorithm groups genes whose similar expression patterns suggest coordinated participation in one or more biological processes. To analyze plumbagin effects on gene expression, we applied enrichment analysis to each k-means group for terms describing gene function. These analyses were performed using Genetic Information Relationship Network (GIRN), which facilitates enrichment analysis based on data consolidated from many other sources, described in (10.1101/079590). The data discussed herein can be explored using a version of GIRN customized to this study (www.voxvill.org/relnet.plumbagin). GIRN uses lists of genes, such as those contained within one or more of the k-means groups of Fig. 2, as seed lists to identify enrichment of parts of the global protein-protein and transcription factor-target gene network in the seed list, and presents the enriched sub-network, with attached annotations, as a force directed subgraph of the global protein-protein and transcription factor-target gene interaction network. The subgraph is enriched insofar as genes not in the seed list are included if they are connected to genes in the seed list more often than would be expected by chance, indicated by a z-score^26^. Genes in the subgraph were then explored for enrichment of associated functional annotation terms. We iterated through the various annotation capabilities of GIRN using a set of common parameters (z-score = 50) to generate networks that are annotated with enriched GO, Pathway, GSEA, Diseases, and Drugs for most of the 36 k-means groups. Three versions of these systematic analyses, Annotations, which contains all genes and annotation terms for each group with p-values or false discovery rates, Summary Annotations, which contains only the top annotations and statistics, and Short Summary Annotations, which contains the top 5 annotations and statistics, can be accessed at www.voxvill.org/Annotations.

### Plumbagin down-regulates many of the genes that are up-regulated by DHT

KM4 (see Fig. 2) contains 73 genes that were up-regulated by treatment with DHT overnight, but sharply repressed by plumbagin, despite the presence of DHT, consistent with plumbagin inhibition of androgen receptor (Ar) activation by DHT, although a post-transcriptional mechanism remains possible. Genes in this group include Calreticulin (Calr), which inhibits the binding of Ar and other nuclear receptors to their binding sites in DNA^40,41^; acetyl-CoA carboxylase (Acaca), which is rate limiting for fatty acid synthesis^42^; arrestin-1 (Arrb1), which is interesting because it may promote tumor survival by metabolic reprogramming to rely on glycolysis, rather than on oxidative phosphorylation, the former favoring the frequently encountered hypoxic tumor environment^43^; and annexin-6 (Anx6), which is over-expressed in aggressive pancreatic cancer. KM4 also contains the androgen receptor (Ar), itself, suggesting that plumbagin may affect prostate cancer by directly down-regulating Ar, which is consistent with our previous observation that plumbagin causes reduction of Ar protein^22^. Down-regulation of these and other functions associated with genes in KM4 by plumbagin may account for some of its anti-tumor properties in a manner similar to ADT. KM4 is enriched for GO biological processes “regulation of endothelial cell migration” and “circulatory system development” (FWER < 0.05) (www.geneontology.org). When KM4 is used as a seed for an enriched network in GIRN, using z-score = 50, the resulting network, which contains a larger number of genes than in KM4, is not enriched for any GO, Pathway, or GSEA term. GIRN generates networks beginning with statistical inferences of nearest neighbor genes. Often, a network including only nearest neighbors does not penetrate sufficiently deeply into the global network to include a significant number of genes associated with any annotation term. To remedy this, the seed list augmented with genes that are inferred as nearest neighbors can be reintroduced to GIRN to extend to the next-nearest neighbors. An augmented seed list for KM4 was produced in this manner, and found to be enriched for GO terms relating to regulation of the extracellular microenvironment, with good false discovery rates (FWER < 0.05) (Table 1). These inferences were due primarily to Bmp4 and inferred genes. Bmp4 promotes prostate tumor growth in bone^44^, implying that the inferred GO terms related to Bmp, Tgf-beta, Smad, and ossification may be relevant plumbagin’s anti-androgen and anti-tumor effects. Zhang *et al*.^45^ showed that inhibition of Plk1 with volarsertib (BI2536) not only inhibits the growth of CRPC in xenografts, but also down-regulates the expression of Ar; down-regulation of Plk1, which is in KM30, may account for down-regulation of Ar and other genes in KM4.

**Table 1:** KM4 next-nearest neighbors are enriched for these GO terms.

GO Term	Primary Link	FWER
BMP receptor binding	GO:0070700	4.15E-10
skeletal system development	GO:0001501	3.79E-8
extracellular space	GO:0005615	3.65E-7
heparin binding	GO:0008201	4.33E-5
BMP signaling pathway	GO:0030509	7.94E-5
transforming growth factor beta receptor binding	GO:0005160	0.0003
positive regulation of pathway-restricted SMAD protein phosphorylation	GO:0010862	0.0006
negative regulation of insulin-like growth factor receptor signaling pathway	GO:0043569	0.001
SMAD protein signal transduction	GO:0060395	0.005
proteinaceous extracellular matrix	GO:0005578	0.007
ossification	GO:0001503	0.015
cytokine activity	GO:0005125	0.015
regulation of complement activation	GO:0030449	0.017
negative regulation of BMP signaling pathway	GO:0030514	0.020
regulation of pathway-restricted SMAD protein phosphorylation	GO:0060393	0.020
positive regulation of DNA-dependent DNA replication	GO:2000105	0.020
cartilage development	GO:0051216	0.049

### Genes that are up-regulated by plumbagin only in the absence of DHT

*In vivo* experiments indicate that regression of PTEN-P2 tumors during plumbagin treatment requires both plumbagin and ADT^6^. KM10, 15, and 36 show responses to plumbagin that are dampened by DHT, and KM31 contains genes that are up-regulated by plumbagin, and down-regulated when both plumbagin and DHT are present. KM31 genes are enriched for GO terms “cell division”, “mitotic cell cycle”, “DNA replication”, and “DNA repair”, and includes cyclin B1, the expression of which decreases in response to plumbagin in several cancers^9,11,46–50^. KM15 is enriched for “response to ethanol”, “gene expression” and “membrane raft”, none of which is particularly informative. However, KM36 is enriched for “exosome (RNase complex)”, “RNA phosphodiester bond hydrolysis, exonucleolytic”, “rRNA processing”, “3′−5′-exoribonuclease activity”, and “nuclear exosome (RNase complex)”, suggesting altered RNA metabolism, discussed later.

Genes in KM10 are largely transcriptionally regulated by Ar, and may mediate events that are induced by plumbagin but suppressed by DHT (Supplementary Figure 1). Using z-score = 50, Atr is inferred. Atr is a serine-threonine kinase involved in the DNA damage repair response, in which it activates a DNA damage checkpoint. Atr phosphorylates numerous genes involved in checkpoint activation, including Chk1, Rad9, Rad17, Brca1, Chek1, Mcm2, Rpa2, Smc1, and Trp3. Atr is in KM31, which is similarly up-regulated by plumbagin in the absence of DHT, but in fact down-regulated in the presence of DHT. So, Atr interacts with genes in KM10 and is a member of KM31. The implication is that Atr induction of apoptosis may be suppressed by androgen, or that cells deprived of androgen are more sensitive to DNA damage.

In KM10 enrichment analysis, only “protein-DNA complex” reaches statistical significance, and no Pathway terms are significant. However, analysis by GSEA indicates that genes from two studies, one specifying the gene set designated MILI_PSEUDOPODIA_HAPTOTAXIS_UP, which contain genes that produce RNAs that localize to protruding pseudopodia in response to migratory stimuli^51^ and one specifying genes involved in the DNA repair response of cells exposed to UVC, designated DACOSTA_UV_RESPONSE_VIA_ERCC3_DN^52^.

**Figure 3:**
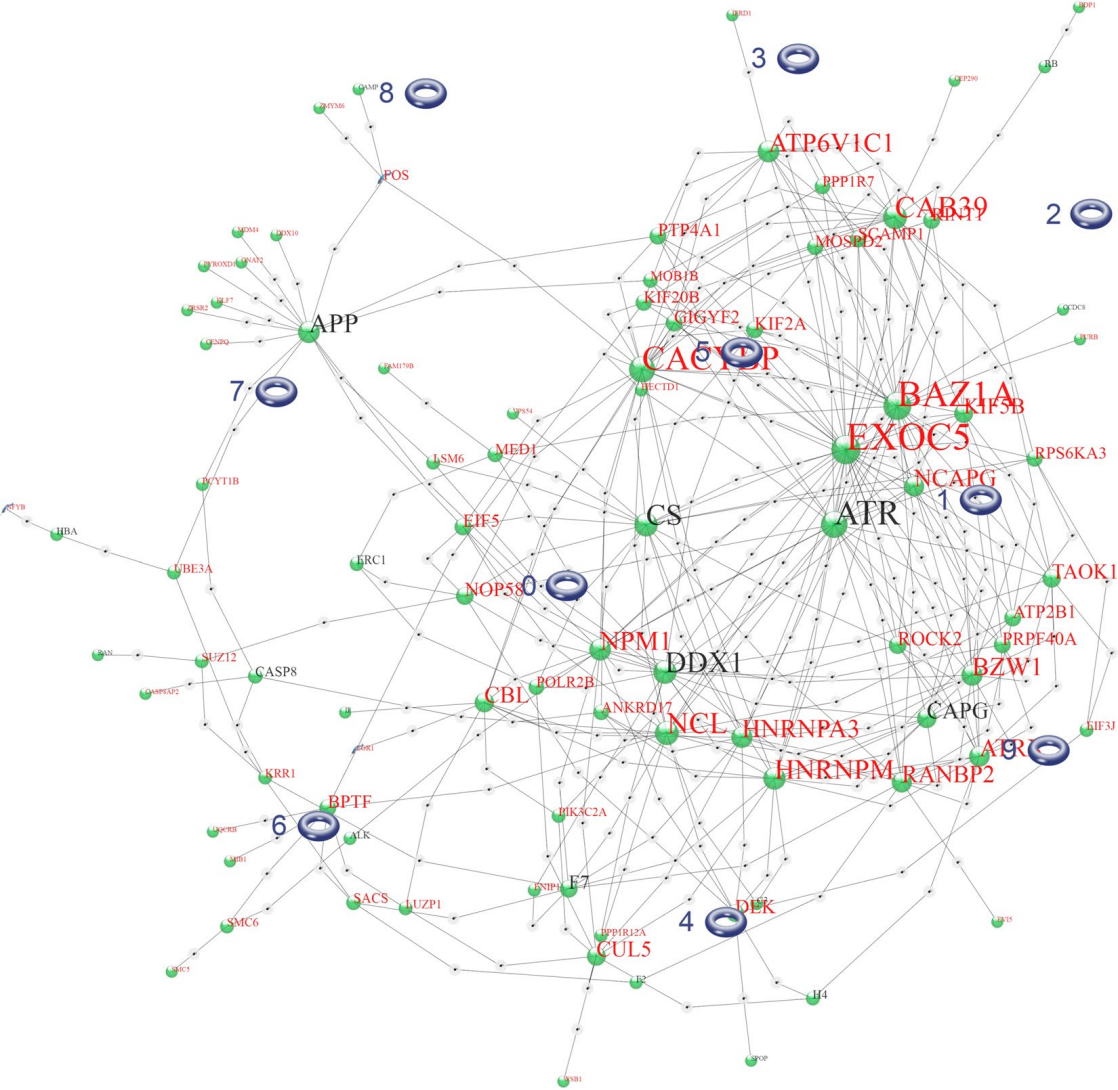
KM10 further parsed into genes that become co-localized due to shared connections in the force-directed graph. Ar was omitted for clarity. Parsing is achieved in GIRN using a utility that groups genes according spatial proximity using k-means.

**Figure 4:**
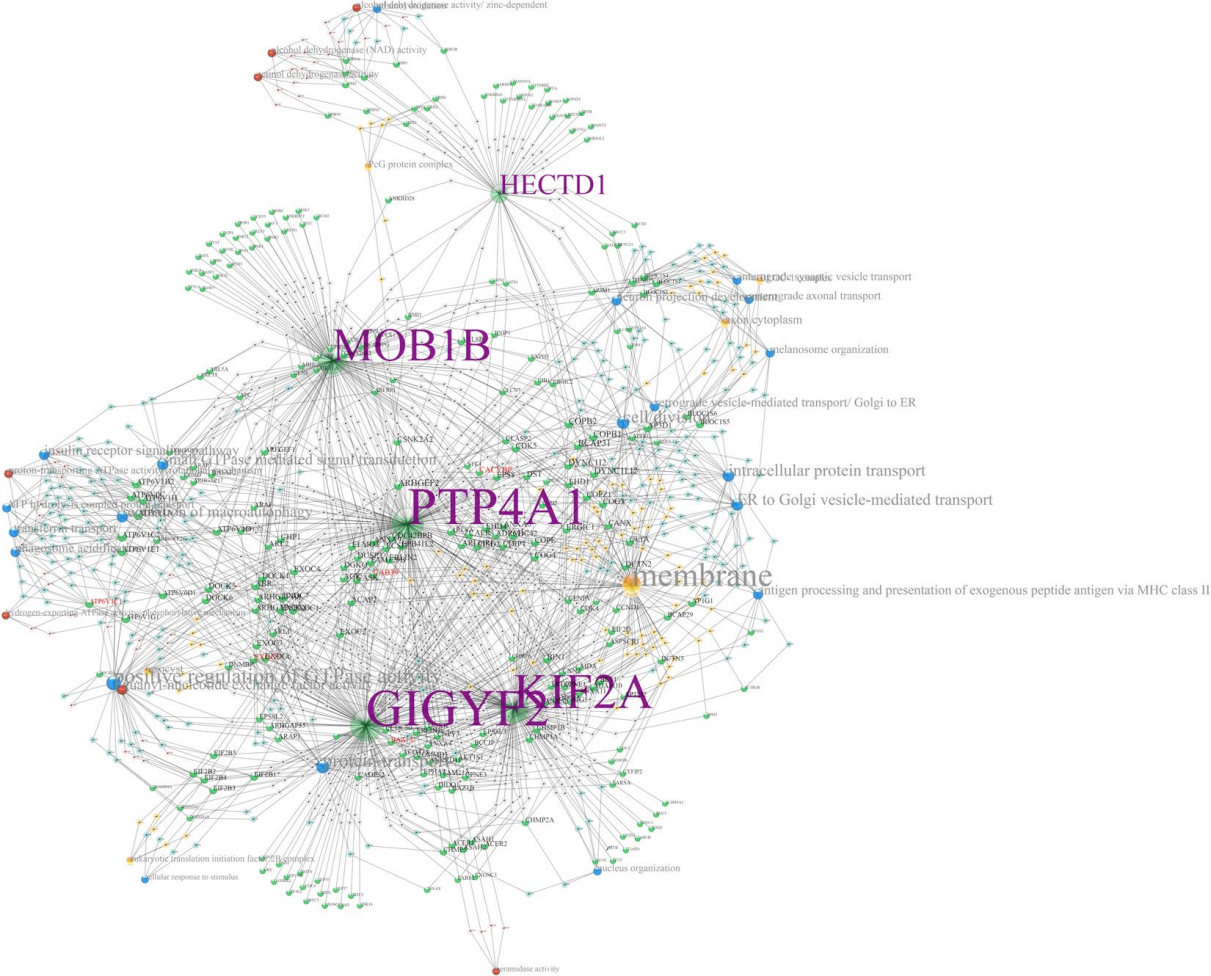
Centroid 5 (shown in Fig. 3) genes were used as a seed in GIRN using z-score = 50 to generate this graph. Enriched Gene Ontology terms were determined.

The strategy of grouping genes by expression profile and testing for enrichment has limitations. One such limitation becomes apparent when two or more biological processes involving different genes are coordinately regulated, and enrichment of terms associated with one group of genes is diluted by the presence of other groups. In force-directed graphs, genes that interact with many of the same genes coalesce into clusters. The network derived from KM10 has evident clusters of genes, indicated in Fig. 3 by small tori, which were placed automatically by a clustering algorithm in GIRN. Ar has been omitted for clarity, but in fact targets 54 of the 94 genes represented in the graph. Centroid 5 contains the genes Gigyf2, Hectd1, Kif2a, Mob1b, and Ptp4a1. Using these genes as seed genes in GIRN with GO term enrichment, the sub-network shown in Fig. 4 is obtained. Supplementary Table 1 contains a list of the statistically significant GO terms (FWER ≤ 0.05) related to membrane transport, including “phagosome acidification”, “anterograde synaptic vesicle transport’, “transferring transport”, “membrane”, “exocyst”, and many others related to this theme. “Cell division” and several other processes are also well represented. Mob1b regulates spindle pole body and mitotic checkpoint processes. The protein-protein interaction network containing these genes is very highly interconnected, which can be seen most clearly prior to attachment of enriched GO terms (Supplementary Figure 2).

Centroid 9 comprises the genes Baz1a, Exoc5, Kif5B, NcapG, Rps6ka3, Scamp1, and Taok1. Using these genes as seeds and annotating with Gene Ontology terms (FWER ≤ 0.05), the DNA replication and repair role of this small module is evident in the terms “meiotic DNA recombinase assembly”, “Rad51B-Rad51C-Rad51D-XRCC2 complex”, “strand invasion”, “recombinase activity”, “double-strand break repair via homologous recombination”, “mitotic recombination”, “four-way junction DNA binding”, and “replication fork” (Supplementary Figure 3). These effects are all due to enrichment of KM10 in genes whose protein products interact with Atr, which does not appear in the original differentially regulated list.

### Genes up-regulated by plumbagin regardless of the presence or absence of DHT

**Figure 5:**
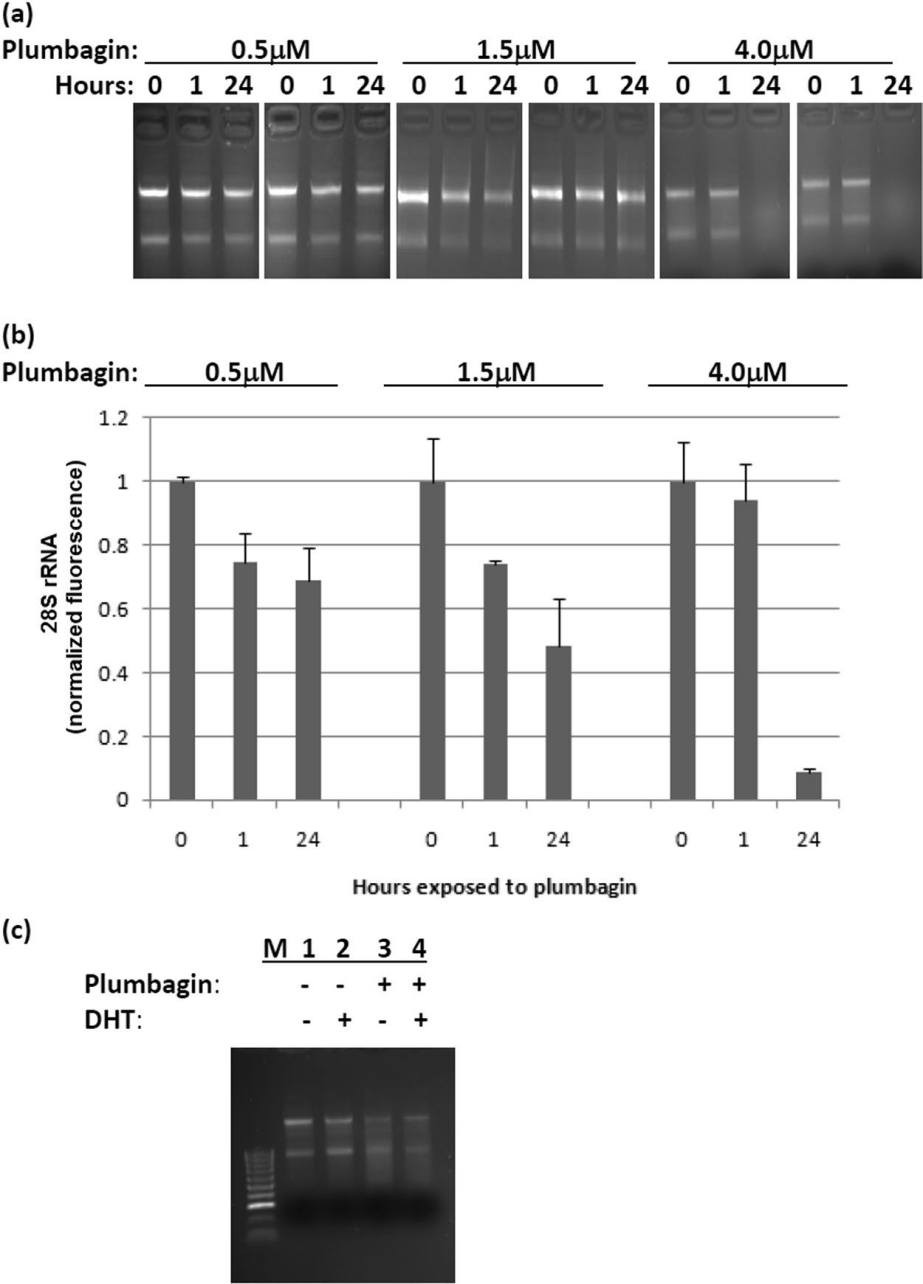
Plumbagin treatment causes RNA degradation. (**a**) Total RNA from PTEN-P2 cells was isolated 0, 1, or 24 hr. after treatment with 0.5, 1.5, or 4.0 μM plumbagin and resolved on agarose gels. The two bands correspond to the ribosomal RNAs large and small subunits. (**b**) Bar graph of ethidium bromide fluorescence of large ribosomal subunit rRNA showing that plumbagin causes RNA degradation over time in proportion to the plumbagin concentration. Error bars represent one standard deviation, n = 2. (**c**) RNA from PTEN-P2 cells exposed to plumbagin at 1.5 μM for 25 hr, or DHT (10^−8^ M) for 48 hr or both.

KM16 contains genes for which transcript abundance increases in response to plumbagin regardless of the presence or absence of DHT. The top 3 gene ontology terms associated with this group pertain to RNA, including “RNA binding”, “termination of RNA polymerase II transcription” and “polysome”, and genes in this group include, for example, Ddx5 (DEAD-box helicase 5), Eif4a2 (eukaryotic translation initiation factor 4a2), Fcf1 (rRNA-processing protein), Fxr1 (Fmr1 autosomal homolog 1), Hnrnph1 (heterogeneous nuclear ribonucleoprotein H1), Magohb (mago homolog B, exon junction complex core component) and 40 others (out of 136 in the z-score = 50 network) annotated as RNA binding proteins by the Gene Ontology Consortium. These terms, together with the known ability of plumbagin to generate reactive oxygen, suggested that genes in KM16 may respond to RNA damage, or disruption of some step in RNA metabolism. The trajectory of KM26 is similar to that of KM16, and like KM16, KM26 is concerned with nucleic acids, including the following top 7 GO terms under Bonferroni correction: “5′-nucleotidase activity”, “purine nucleotide catabolic process”, “pyrimidine nucleoside catabolic process”, “nucleobase-containing small molecule metabolic process”, “pyrimidine nucleobase metabolic process”, “purine nucleobase metabolic process”, and “adenosine metabolic process”. Therefore we examined ribosomal RNA by gel electrophoresis and found that 24 hr treatment with plumbagin causes damage to ribosomal RNA. Although >20% of the ribosomal RNA in PTEN-P2 cells is damaged after 24 hr treatment with 0.5–1.5 μM plumbagin (Fig. 5), this damage surprisingly does not severely inhibit cell division (compare Fig. 1). Mechanisms that damage rRNA may also damage mRNA. In Fig. 2, some decreases in mRNA may be attributable to such a mechanism. However, the magnitude of decrease in Fig. 2 is often considerably larger than that observed for 0.5 μM plumbagin in Fig. 5b. Also, DHT does not protect cells from plumbagin-induced ribosomal RNA damage (Fig. 5c). Additional gel pictures are available in Supplementary Figure 5.

### Plumbagin and DHT down-regulate cell cycle control genes cooperatively

**Figure 6:**
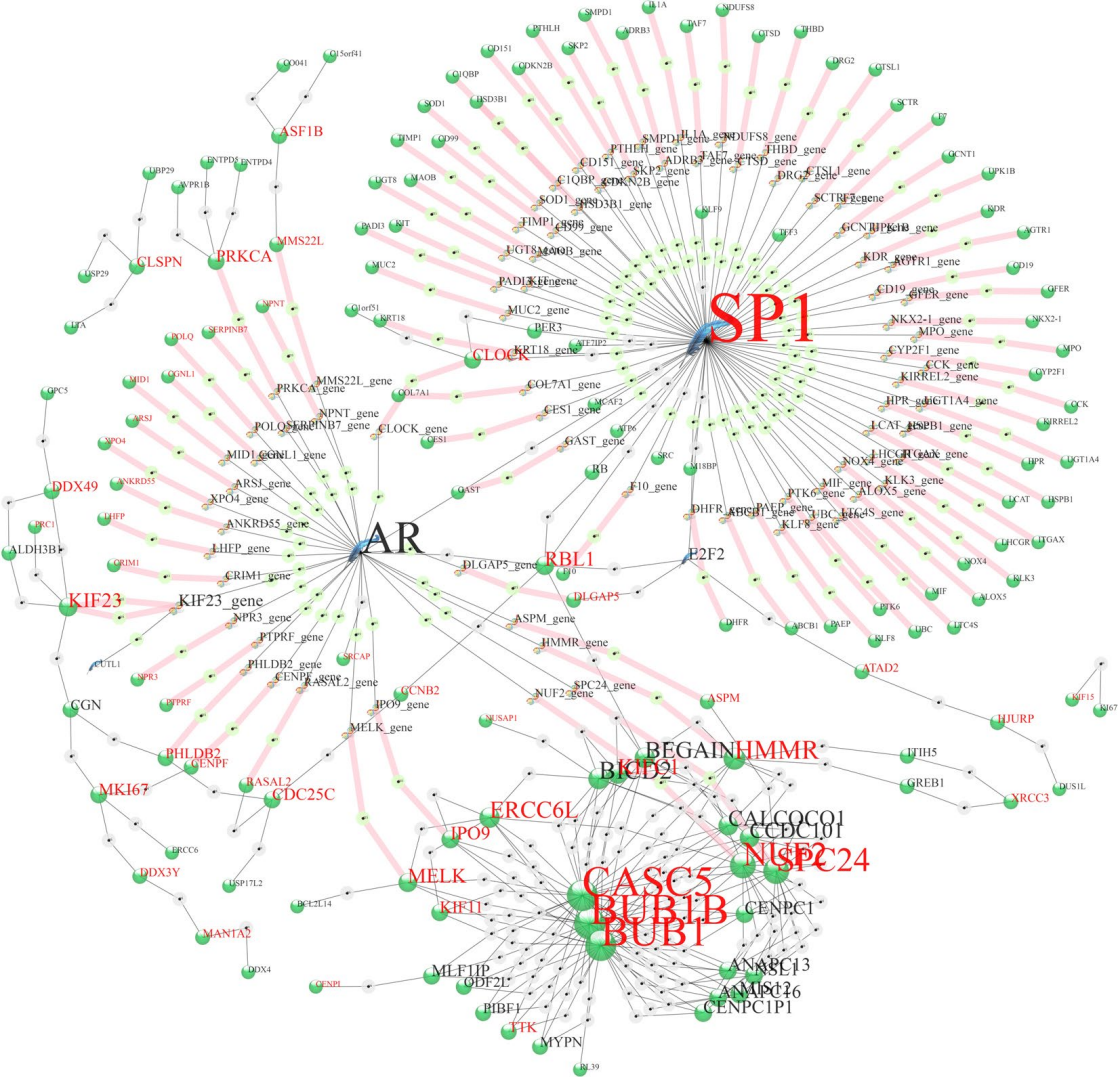
KM5 rendered by GIRN. Red font indicates genes in KM5, black font indicates genes inferred from the global network at z-score = 50. For example, SP1 is a member of KM5, whereas Ar is inferred. The Quill icon represents transcription factors, the Alpha-Helix icons represent genes, the Green Marble icons represent proteins, the gray and green icons represent information nodes from GIRN. The thick pink lines connect genes and their corresponding proteins.

KM5 is one of several groups in which cells respond to plumbagin through an androgen-mediated pathway. A force-directed graph from GIRN for KM5 is shown in Fig. 6. In this group, plumbagin sharply down-regulates the transcripts of genes when DHT is present but has little or no effect when DHT is absent. Unlike KM4, these genes are not induced by DHT. KM5 is one of several groups in which a DHT-responsive mechanism represses genes when activated by plumbagin. It is unknown whether this decrease in mRNA abundance is due to cessation of transcription, accelerated decay, or both. Genes in this group control mitosis by mediating spindle attachment to kinetochores. The GO terms significantly enriched in this group comprise “mitotic nuclear division”, “mitotic cell cycle”, “cell division”, “chromosome segregation”, “condensed chromosome kinetochore”, “mitotic spindle assembly checkpoint”, “small GTPase mediated signal transduction”, “cell proliferation” and “spindle”, using Bonferroni correction (FWER ≤ 0.05). Group members Nuf2, Casc5 (Knl1), and Spc24 are all components of the kinetochore, whereas Bub1 and Bub1r are members of the spindle assembly checkpoint (SAC) protein complex^53^. Bub1 and Ttk(Mps1) are essential SAC kinases. Ercc6l is Ercc excision repair 6 like, spindle assembly checkpoint helicase^54^; the kinesins Kifll (Eg5), Kif15, Kif23, and Kifc1 function to regulate microtubule dynamics during cell division^55,56^. Pathway enrichment includes several that overtly related to cell division, as well as “plk1 signaling events” (from PID and NDEx), which is a prominent regulator of mitosis and cytokinesis^57^. Plk1, itself, is a member of KM30, which is almost identical in trajectory to KM5. Plk1 is down-regulated by plumbagin without DHT, but more strongly down-regulated by the combination of plumbagin and DHT. BIRC5 (survivin) is also in KM30; the genes Aurkb, Foxm1 and Incenp are in KM28, which has a similar profile; and Gsg2 (haspin) is in KM29, which contains genes that are down-regulated by plumbagin regardless of DHT. All of these genes, Birc5, Aurkb, Foxm1, Incenp, and Gsg2 are important in centromere structure and dynamics^57^. Prkce and Stat3 been reported to be down-regulated in PTEN-KO (i.e. knock-out) mice treated with plumbagin^58^. Neither Prkce nor Stat3 are expressed in PTEN-P2 cells, but Prkca is in KM5, while several Stat family members, Stat1, 6, and 5a, are in KM15, which contains genes down-regulated by plumbagin, Stat5b is in KM28, which has a similar profile to KM5, and Stat2 (KM36) is down-regulated by plumbagin in the absence of DHT. Also, similar to PTEN-KO mice^58^, Cox-2 (aka Pptgs2; KM30) is down-regulated in PTEN-P2 cells when treated with plumbagin. Plumbagin and COX-2 inhibitor celecoxib act synergistically in melanoma to induce apoptosis^59^.

No gene ontology terms are enriched in KM30 when a Bonferroni adjustment is applied. Nearest neighbors, however, are enriched for “spindle midzone”, consistent with properties of KM5. Although not statistically enriched in KM30, the top 7 enriched terms are, sequentially by p-value, “spindle midzone”, “regulation of attachment of spindle microtubules to kinetochore”, “positive regulation of DNA endoreduplication”, “spindle pole”, “sister chromatid cohesion”, “midbody”, and “mitotic cytokinesis”. One of the reasons that GIRN misses apparent enriched functions such as these is that multiple functional groups are inevitably included in the same k-means group, thereby diluting their effect on the p-value in Fisher’s exact test. In fact, while term enrichment is meaningful, the correct null hypothesis is that alteration of the activity of a single gene, e.g. phosphorylation or differential expression can, in principle, alter a biological process. In GIRN, a group of genes mapped to the global network can be further divided into those that co-localize spatially in the graph due to shared neighborhoods with other genes; co-localization of genes in similar neighborhoods is a property of force directed graphs. GIRN uses k-means to identify co-localized genes, designated by centroid number. When KM30 is further divided into 5 colocalized subgroups, centroid-1 consisting of the seed genes Aurka, Axl, CA2, Ccnb1, Cdc20, Csk, Ect2, Epha2, Kif2c, Mt2, Nsl1, Slfn11, and Tln1 plus genes inferred from the network, “cell division” is inferred as the most significant term under Bonferroni correction. Centroid-2 from KM30, using Htr1f, Abi3, Abi3bp, Ada, Adam12, Arl4d, Cav1, Cd4, Cd44, Eps8, Fbln5, Fbn1, Flrt1, Flrt2, Gja3, Hbegf, Htr1f, Igfbp3, Ltbp2, Madcam1, Neu3, Neu4, Pou3f3, Ptgs1, Ptgs2, Ndrg1, Rab27b, Sdpr, Sgcd, Tead4, Tex28, Tex28p2, and Vgll4_HUMAN.YAP1 as seed genes, is heavily enriched in GO terms related to extracellular matrix under Bonferroni correction (FWER ≤ 0.05), including “extracellular matrix organization”, “caveola”, “membrane raft”, and others. KM28 is similar to Groups 5 and 30, and is enriched for the GO terms “DNA replication” and “mitotic cell cycle” (as well as “liver development” and “response to estradiol”) under Bonferroni correction (FWER ≤ 0.05), suggesting that down-regulation of KM28 genes favors cell cycle arrest. However, treatment with 0.5 μM plumbagin in the presence of DHT is quite effective in reducing the transcripts of these genes without causing cell cycle arrest *in vitro* (see Fig. 1), suggesting either that the spindle assembly proteins, which are abundant, may be sufficient to support more than one round of cell cycle, or that suppression of their transcription is transient relative to our 1 hr window.

### Plumbagin regulates androgen receptor target genes disproportionately

One frequently seen gene expression response is down-regulation in the presence of both plumbagin and DHT, but no decrease in the presence of either agent alone (KM5, 20, 21, 28, 30, 31). This suggests that the cellular response to plumbagin is mediated by Ar. If this were true, Ar transcriptional target genes may be enriched in these groups. However, this is not certain. Alternatively, the response could be due to one or more regulators that are indirectly affected by DHT, for example, a transcription factor or signal transduction molecule that is regulated by androgen receptor, or by DHT independently of Ar. Therefore, we determined whether Ar target genes are enriched in KM5, 20, 21, 28, 30, and 31. GIRN allows one to determine the number of genes in a seed list that are regulated by several well-studied transcription factors, including Ar, Ets1, Gata1, Gata2, Ybx1, Foxp3, E2f4, and others. Indeed, 5 out of 6 of these KM groups taken individually are enriched for androgen receptor target genes, with p ≤ 0.05 for KM20, 21, 28, 30, and 31, but not KM5. Collectively, KM5, 20, 21, 28, 30, and 31 contain 627 Ar target genes out of 1429 genes (i.e. 44%), whereas, globally, Ar binds to the promoters of 6443 of 23192 genes (28%). The Fisher’s exact test p-value indicating enrichment is p = 2.2 × 10^−16^, but there are 1107568 possible combinations of 33 KM groups (excluding sparse groups KM2, 3 and 6) taken 6 at a time, so the Bonferroni-corrected p-value is p = 2.4 × 10^−10^. Determination of the p-values for five thousand (5000) randomly selected groups of 6 KM groups, i.e. permutation analysis, yielded only one smaller p-value (i.e. 0.02%). This means that genes that are down-regulated by plumbagin in the presence of DHT are enriched for genes for which Ar is a transcription factor. Interestingly, Esr1, a member of KM28, is not a known Ar target gene, but does engage in protein-protein interactions with Ar. This may indicate that Ar can regulate genes that are not direct transcription factor targets of Ar^60^.

Another transcription factor that appears frequently with Ar is the Ets1 transcription factor, which interacts with the promoters of ~70% of Ar target genes, and may interact physically with Ar^61^. In the composite group consisting of KM5, 20, 21, 28, 30, 31, Ets1 is enriched relative to the ensemble of all genes with a p-value of p = 6.8 × 10^−40^, with Bonferroni correction. However, permutation analysis indicated that 1932 out of 5000 (39%) of randomly selected groups of six KM groups resulted in smaller corrected p-values. This means that Ets1 target genes were enriched by the original selection criteria used to include genes during k-means analysis. Those criteria included (1) responsiveness to DHT, or (2) responsiveness to plumbagin, or both, and (3) an average of at least one FPKM when all FPKM per condition were averaged. Therefore, these data provide evidence that Ar plays a role in the down-regulation due to plumbagin observed in groups KM5, 20, 21, 28, 30, and 31, but that the role of ETS1, if any, is not clear.

In KM16 and 26, in which plumbagin causes up-regulation of 360 genes without any apparent effect of DHT, 89 out of 314 genes (28%) are targets of plumbagin, approximately the global average for Ar targets. Therefore, the expression behavior of these genes is not expected to be attributable to Ar.

### Genes that are down-regulated by plumbagin regardless of DHT

KM15, 24, and 29 comprise genes that are down-regulated by plumbagin, without small or no DHT affects. Theoretically, genes that display this behavior could synergize with genes that are responsive to DHT to explain the *in vivo* synergism between plumbagin and DHT. KM15 is concerned with “neutrophil degranulation”, “secretory granule lumen”, “aging”, and “ficolin-1-rich granule lumen”. “Extracellular space” and “extracellular matrix organization” are invoked by the genes in KM24. Ar targets 29%, 30% and 33% of the genes in KM15, 24, and 29, which are unremarkable relative to the global average of 28%.

## Discussion

Plumbagin is a potential chemotherapeutic for treatment of various cancers, including prostate cancer. In the studies described herein, we examined the effects of plumbagin on gene expression in a murine prostate cancer cell line that we used previously to generate experimental tumor models for prostate cancer^6,22^. In those studies, we observed that plumbagin synergizes with androgen deprivation to cause tumor regression. Here, we used RNA-seq and bioinformatic methods to gain a better understand the cellular response to plumbagin, and possibly gain insight into the molecular mechanisms that might account for the synergistic interaction between plumbagin and DHT.

We found mild, but reproducible, synergy between plumbagin and DHT with regard to cell growth *in vitro*. However, we found substantial changes in gene expression that differed with respect to treatment with plumbagin, DHT, or both. In these experiments, RNA was purified after only one hour of treatment with plumbagin, in an attempt to minimize secondary responses. A surprising result was that plumbagin treatment in combination with DHT appears to activate a repressive phenomenon that affects genes that are regulated by Ar disproportionately. The molecular basis for this phenomenon is unknown, but the enrichment of Ar target genes in this response class suggests possible modification of the Ar itself, or modification of a repressive complex in which it plays a role. This repressive phenomenon cannot easily explain our previous observation that plumbagin and ADT synergize to cause regression of experimental tumors. In the absence of DHT, these repressive phenomena are not observed.

Plumbagin alone can partially arrest prostate tumor growth, and ADT can arrest the growth of androgen-dependent PTEN-P2 tumors *in vivo*, but the combination was required to cause tumor regression^6^. Differential regulation of genes that depend on both plumbagin and DHT would seem not to pertain to the requirement that ADT and plumbagin treatment be combined to cause tumor regression. The observation that some metastatic prostate tumors produce androgens intratumorally may be relevant^62–65^. In this scenario, androgen production within the tumor could support gene expression dependent on both plumbagin and androgen, while the tumor microenvironment experiences androgen deprivation. In PTEN-P2 cells growing in culture, steroidogenic enzymes Cyp17a1 and Hsd3b1 were undetectable by RNA-seq, but their status *in vivo* is unknown.

There are several response classes consistent with the *in vivo* synergy of plumbagin and ADT. In some, plumbagin up-regulates or down-regulates genes independently of DHT, and it is possible that the cytostatic effect of ADT combines with a cellular response to plumbagin to lead to cell death, whereas neither the ADT nor plumbagin treatment alone is sufficient. One such affect may be that of plumbagin on RNA integrity. This possibility was suggested by the enrichment of GO terms concerning RNA metabolism in KM16 and 26. Plumbagin, even at concentrations well below the IC50 for PTEN-P2 cells in culture, causes extensive RNA damage independently of DHT. Surprisingly, despite damage to as much as 50% of ribosomal RNA, the cells continue to divide. It is not yet clear how much RNA damage occurs *in vivo* or whether in combination with ADT, it would lead to cell death, or preferentially to the death of tumor cells.

The difference noted between *in vitro* and *in vivo* effects of DHT on plumbagin treatment also suggest that ADT may synergize with plumbagin via tumor microenvironment factors that were not present in the *in vitro* experiments. Detailed examination of gene expression in resected experimental tumors from animals exposed to combinations of plumbagin and ADT might further shed light on the pathways involved.

## Electronic supplementary material

Supplementary Data
