# Supplementary material for: Differential gene expression induced by anti-cancer agent plumbagin is mediated by androgen receptor in prostate cancer cells
Source: Sci Rep. 2018 Feb 9;8:2694. doi: 10.1038/s41598-018-20451-9 (PMC5807367; doi:10.1038/s41598-018-20451-9)
Supplement: Supplementary file 1 — Supplementary Data [file 41598_2018_20451_MOESM1_ESM.pdf]

Supplementary Data

Title: Differential gene expression induced by anti-cancer agent plumbagin is mediated by androgen receptor in prostate cancer cells.

Gaelle Rondeau[1], Parisa Abedinpour[1], Adrian Chrastina[1], Jennifer Pelayo[1], Per Borgstrom[1,2], and John Welsh[1]*

[1]Vaccine Research Institute of San Diego, 3030 Bunker Hill Street, Suite 205, San Diego, CA 92109

[2]Pellficure Pharmaceuticals, Inc., 2325 Camino del Collado, La Jolla, CA 92037

*To whom correspondence should be addressed:

jwelsh@sdibr.org

(442) 222-9923

Supplementary Figure 1. The enriched local network containing KM10 genes shows that many KM10 genes are transcriptionally regulated by androgen receptor (Ar). GIRN indicates the functional link between a gene and the protein it encodes with a pink line.

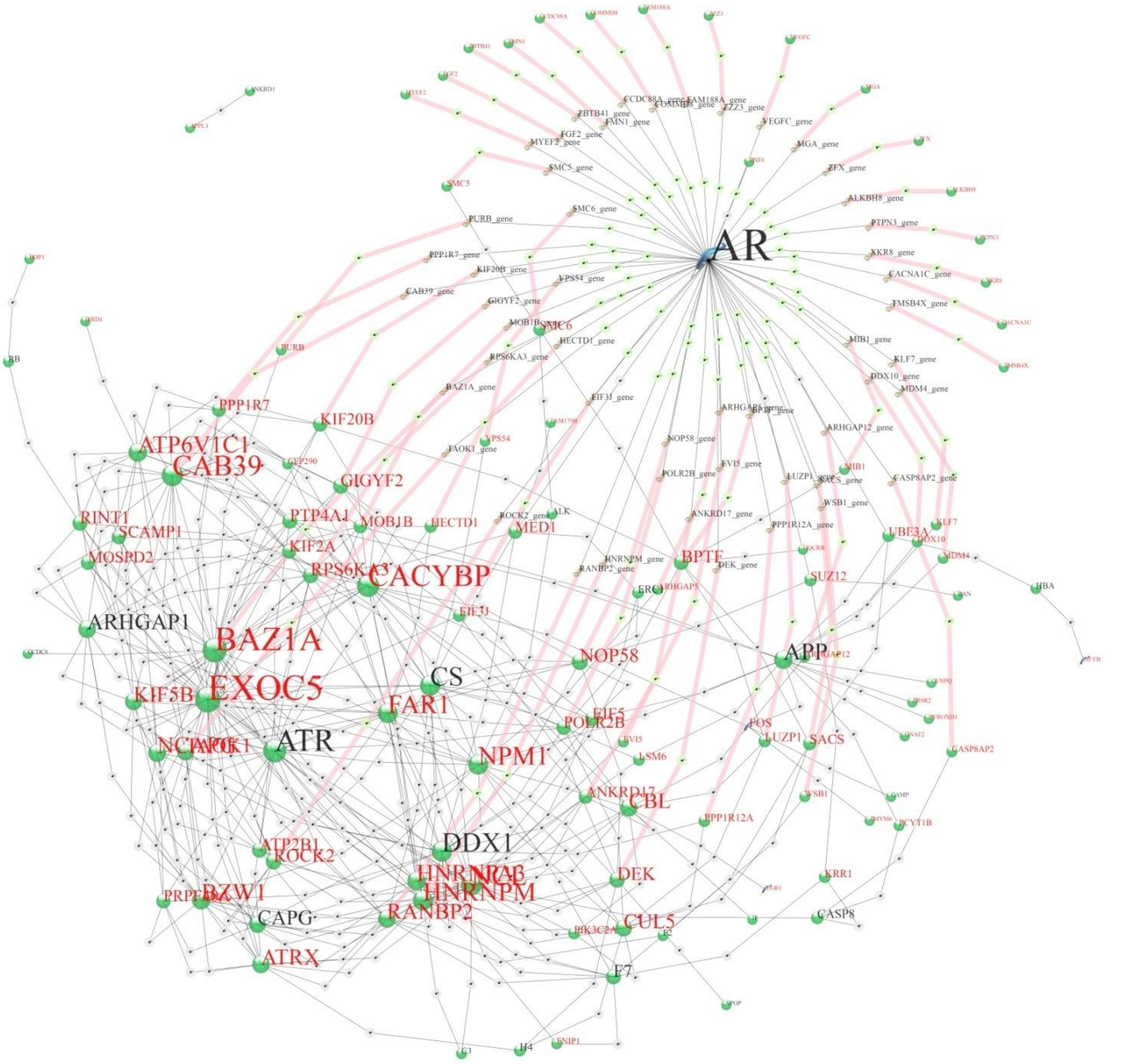

Supplementary Figure 2. Genes Gigyf2, Hectd1, Kif2a, Mob1b, and Ptp4a1 form a spatially colocalized group represented by centroid 5 in **Figure 3**, which illustrates the network around KM10 genes. When these genes are used as an input list in GIRN, they give rise to this highly interconnected network. This contains the same genes as **Figure 4**, without GO terms attached.

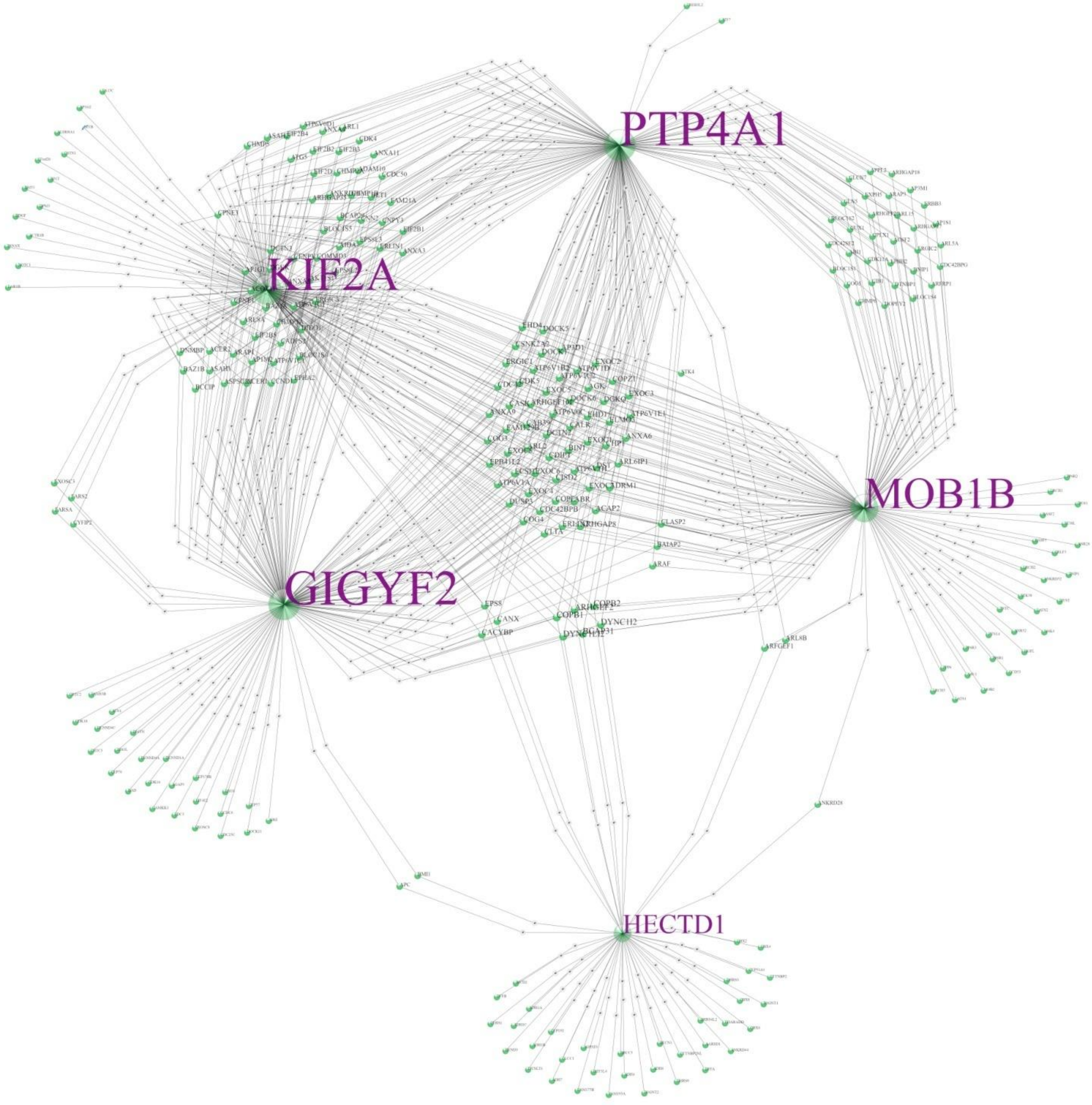

Supplementary Figure 3. Genes from centroid 1, **Figure 3** are enriched for GO terms concerned with DNA damage and repair; Atr is a DNA damage cell cycle checkpoint regulator.

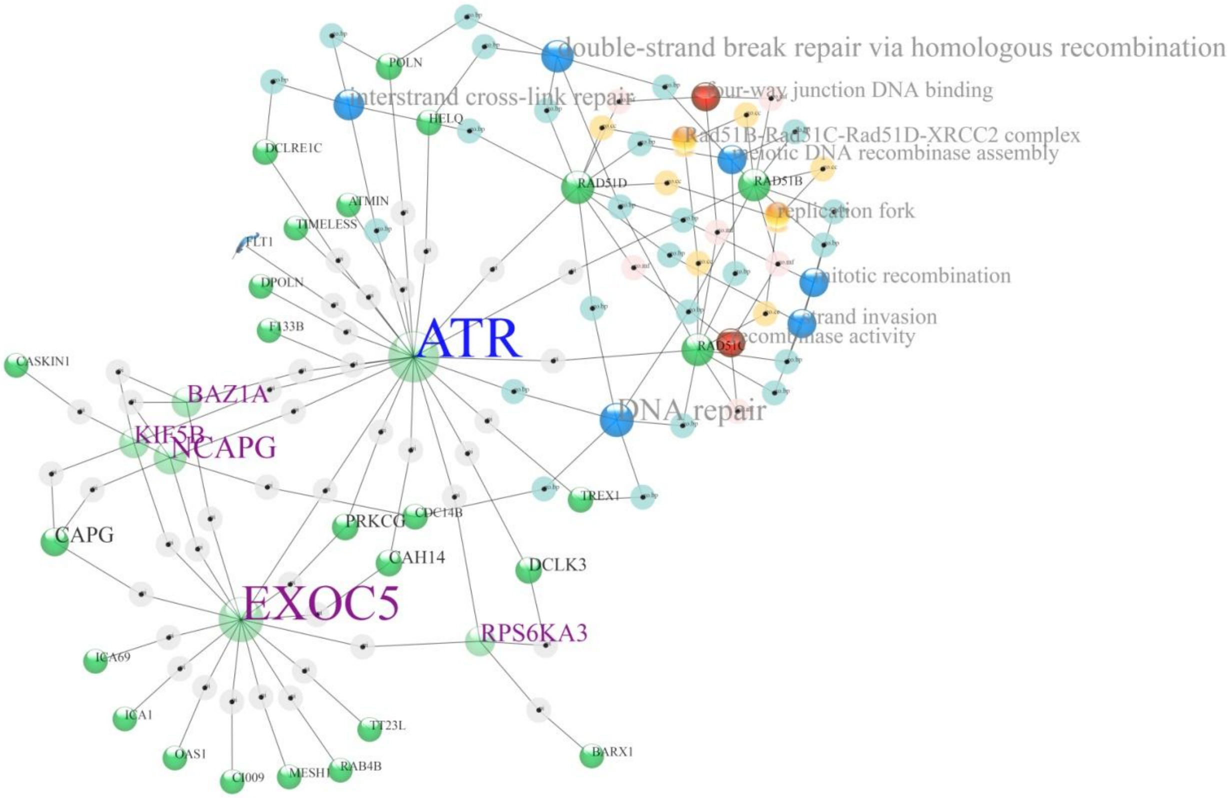

Supplementary Figure 4. Original gel pictures for Figure 5, showing accelerated rRNA decay due to plumbagin treatment. (a) 0.5μM plumbagin, (b) 1.5μM plumbagin, (c) 4.0μM plumbagin. Lanes: 0hr, 1hr, 24hr, 48hr, 72hr, Marker, 0hr, 1hr, 24hr, 48hr, 72hr. Marker is 1μg 1kb Bioline HyperLadder^{TM}.

(a) 0 1 24 48 72 M 0 1 24 48 72hr

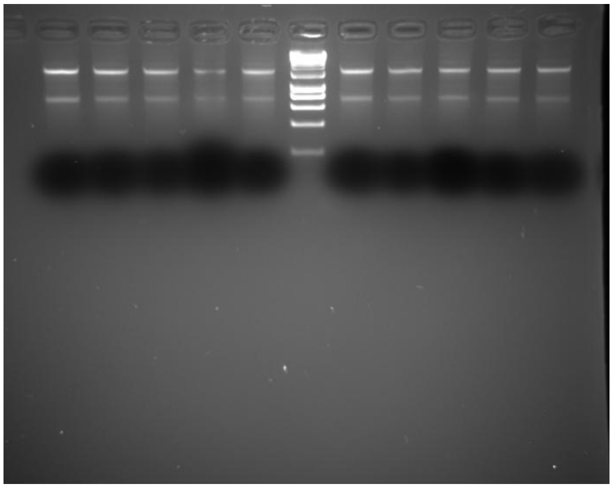

(b) 0 1 24 48 72 M 0 1 24 48 72hr

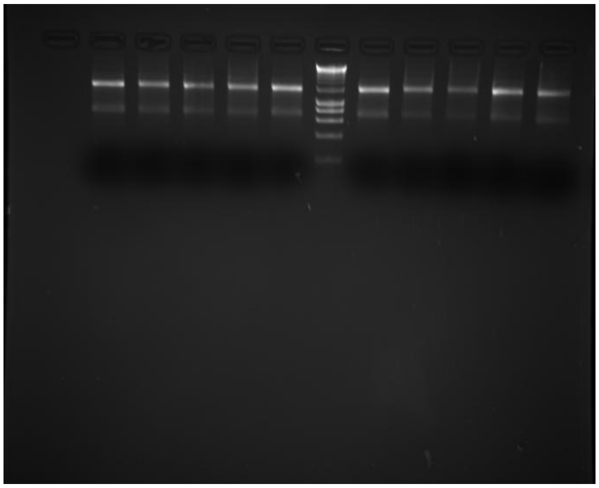

(c) 0 1 24 48 72 M 0 1 24 48 72hr

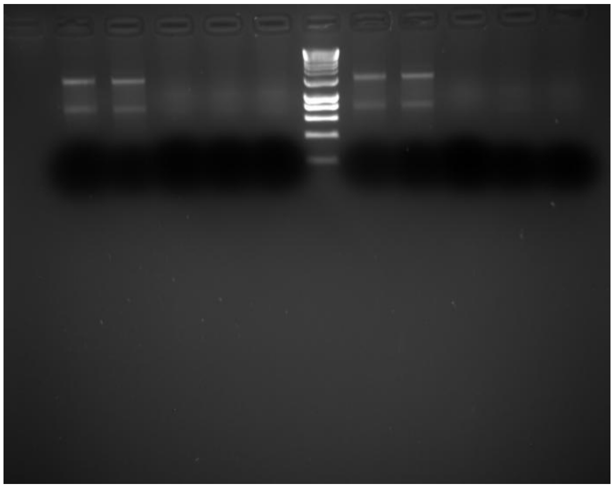

Supplementary Table 1. Gene Ontology Terms enriched in centroid 5 of KM10.

GO Term	Primary Link	FDR
phagosome acidification	GO:0090383	2.17E-8
anterograde synaptic vesicle transport	GO:0048490	6.67E-7
transferrin transport	GO:0033572	1.86E-6
membrane	GO:0016020	2.72E-6
exocyst	GO:0000145	4.65E-6
small GTPase mediated signal transduction	GO:0007264	7.38E-6
alcohol dehydrogenase (NAD) activity	GO:0004022	7.672E-6
melanosome organization	GO:0032438	1.04E-5
regulation of macroautophagy	GO:0016241	1.69E-5
alcohol dehydrogenase activity, zinc-dependent	GO:0004024	1.90E-5
anterograde axonal transport	GO:0008089	2.16E-5
ATP hydrolysis coupled proton transport	GO:0015991	3.04E-5
proton-transporting ATPase activity, rotational mechanism	GO:0046961	5.56E-5
protein transport	GO:0015031	9.93E-5
insulin receptor signaling pathway	GO:0008286	0.0001
hydrogen-exporting ATPase activity, phosphorylative mechanism	GO:0008553	0.0001
ER to Golgi vesicle-mediated transport	GO:0006888	0.0002
intracellular protein transport	GO:0006886	0.0002
cellular response to stimulus	GO:0051716	0.0005
axon cytoplasm	GO:1904115	0.0007
antigen processing and presentation of exogenous peptide antigen via MHC class II	GO:0019886	0.001
ethanol oxidation	GO:0006069	0.002
eukaryotic translation initiation factor 2B complex	GO:0005851	0.003
cell division	GO:0051301	0.005
guanyl-nucleotide exchange factor activity	GO:0005085	0.006
nucleus organization	GO:0006997	0.008
retrograde vesicle-mediated transport, Golgi to ER	GO:0006890	0.01
ceramidase activity	GO:0017040	0.02
positive regulation of GTPase activity	GO:0043547	0.03
retinol dehydrogenase activity	GO:0004745	0.03
neuron projection development	GO:0031175	0.03
BLOC-1 complex	GO:0031083	0.03
PcG protein complex	GO:0031519	0.03
